# Are caffeine effects equivalent between different modes of administration: the acute effects of 3 mg.kg^−1^ caffeine on the muscular strength and power of male university Rugby Union players

**DOI:** 10.1080/15502783.2024.2419385

**Published:** 2024-10-22

**Authors:** Jason Tallis, Michael, J. Duncan, Neil, D. Clarke, Rhys O. Morris, Ryan, A. Tamilio

**Affiliations:** a Coventry University, Centre for Physical Activity, Sport & Exercise Science, Coventry, UK; b Birmingham City University, Research Centre for Life and Sport Science (CLaSS), School of Health Sciences, Birmingham, UK; c University of Birmingham, School of Pharmacy, School of Health Sciences, College of Medicine and Health, Birmingham, UK

**Keywords:** Caffeine gum, caffeine mouthrinse, ergogenic aids, muscular function, skeletal muscle

## Abstract

**Background:**

There is growing interest in the potential of alternative modes of caffeine administration for enhancing sports performance. Given that alternative modes may evoke improved physical performance via distinct mechanisms, effects may not be comparable and studies directly comparing the erogenicity of alternative modes of caffeine administration are lacking. To address this knowledge gap, the present study evaluated the effect of 3 mg·kg^−1^ caffeine delivered in anhydrous form via capsule ingestion, chewing gum or mouth rinsing on measures of muscular strength, power, and strength endurance in male Rugby Union players.

**Methods:**

Twenty-seven participants completed the study (Mean ± SD: Age 20 ± 2 yrs; daily caffeine consumption 188 ± 88 mg). Following assessments and reassessment of chest press (CP), shoulder press (SP), Deadlift (DL), and Squat (SQ) 1-repetition maximum (1RM) and familiarization to the experimental procedures, participants completed six experimental trials where they were administered 3 mg.kg^−1^ caffeine (Caff) or placebo (Plac) capsule_(CAP)_, chewing gum_(GUM)_ or mouth rinse_(RINSE)_ in a randomized, double-blind and counterbalanced fashion prior to force platform assessment of countermovement jump, drop jump and isometric mid-thigh pull performance. Strength endurance was measured across two sets of CP, SP, DL, and SQ at 70% 1RM until failure. Pre-exercise perceptions of motivation and arousal were also determined.

**Results:**

Caffeine increased perceived readiness to invest mental effort (*p* = .038; ηp^2^=.156), countermovement jump height (*p* = .035; ηp^2^=.160) and SQ repetitions until failure in the first set (*p* < .001; d = .481), but there was no effect of delivery mode (*p* > .687; ηp^2^<.015). Readiness to invest physical effort, felt arousal, drop jump height, countermovement jump, drop jump and isometric mid-thigh pull ground reaction force-time characteristics and repetitions until failure in CP, SP and DL were not affected by caffeine administration or mode of caffeine delivery (*p* > .0.052; ηp^2^< .136).

**Conclusion:**

3 mg.kg^−1^ caffeine administered via capsule, gum or mouth rinse had limited effects on muscular strength, power, and strength endurance. Small effects of caffeine on CMJ height could not be explained by changes in specific ground reaction force-time characteristics and were not transferable to DJ performance, and effects specific to the SQ RTP exercise underpin the complexity in understanding effects of caffeine on muscular function. Novel modes of caffeine administration proposed to evoke benefits via distinct mechanisms did not offer unique effects, and the small number of effects demonstrated may have little translation to a single performance trial when data examining direct comparison of each caffeine vehicle compared against a mode matched placebo is considered.

## 1. Introduction

The performance-enhancing potential of acute caffeine ingestion has been firmly established, with several meta-analyses demonstrating benefits for muscular function [1,2], aerobic endurance [3,4], anaerobic power [5], execution of sport-specific skills [6,7] and cognitive functions [8]. The current understanding of caffeine’s effect, and the foundation from which the conclusions of meta-analyses are recommendations to athletes are drawn, is mostly derived from studies that implement ingestion of caffeine anhydrous administered in a capsule or dissolved in liquid. However, recent interest has grown in understanding the acute performance enhancing potential of alternative forms of caffeine administration (e.g. gum, mouth rinsing, dissolvable strips, nasal sprays), many of which have become more accessible, may offer distinct benefits to athletes, and may not be comparable given proposed action via distinct mechanisms.

One mode of administration that has received growing attention is caffeinated chewing gum, which may offer distinct benefits to athletes given rapid absorption and a faster onset of pharmacological effects. Caffeine released from chewing gum due to maceration in the mouth has been suggested to be absorbed into the bloodstream via the highly vascularized buccal mucosa [9]. Furthermore, caffeine may elicit performance enhancing effects via activation of bitter taste receptors [10], which stimulate brain regions associated with information processing and reward [11]. Following ingestion, the performance enhancing effect of caffeine is primarily attributed to its action as a central nervous system stimulant, acting as an adenosine receptor antagonist at A_1_ and A_2a_ subunits, suppressing the adenosine-induced reduction in excitatory neurotransmitters [12]. Evidence suggest that adenosine receptors are prevalent in the oral cavity of mammals [13], which may mechanistically contribute to the ergogenic effect induced by caffeinated chewing gum. Absorption of caffeine in the mouth is supported by evidence indicating an initial spike in blood plasma concentration ~ 10 minutes post chewing followed by a second peak ~ 40 minutes later due to absorption in the gut [14]. Therefore, caffeinated gum may evoke beneficial effects due to both actions in the mouth alongside well-established mechanisms associated with ingestion.

Concurrently, a growing body of evidence indicates that caffeinated chewing gum evokes beneficial effects for endurance performance [15,16], anaerobic power [16,17], muscular strength and power [16,18,19] and cognitive function [20,21]. The effectiveness of caffeinated gum has been summarized in a recent meta-analysis [22], although sub-analysis indicated that effects were only prevalent when exercise commenced within 15 minutes of ingestion and that benefits were specific to trained participants, but were prevalent across both endurance and strength and power activities. However, it should be acknowledged that studies investigating the ergogenic potential of caffeinated gum around the timeframe of the second peak are sparse. One approach to isolate the contribution of mechanisms associated with caffeine’s action in the mouth is to examine the effects of mouth rinsing, where caffeine is not ingested but a low volume high concentration solution is rinsed around the mouth (typically for 2–20 seconds).

Mouth rinsing may be particularly beneficial for athletes, potentially mitigating detrimental side effects reported in some individuals following ingestion [23]. Although the evidence base is less convincing, a small number of studies indicate caffeine mouth rinsing may evoke beneficial effects on endurance activity [24,25], anaerobic exercise [26], muscular strength [27], and cognitive function [28,29]. Specifically a recent systematic review indicated positive effects in only five of 15 studies evaluating physical performance, although many of the studies included had low methodological quality [30]. Caffeine mouth rinsing may hold some performance-enhancing potential and ambiguity in previous work highlights a need for further investigation.

One important knowledge gap pertaining to the effects of different modes of caffeine administration on sports performance is their direct comparison, where equivalent responses should not be assumed due to the potential to evoke effects via distinct mechanisms. Direct comparison is important in allowing athletes to make decisions regarding appropriate caffeine consumption strategy. To date, the comparative effects of different modes of caffeine administration on physical performance has only been considered in two recent investigations. Whalley, Dearing and Paton [31] demonstrated comparable performance enhancing effects of 3–4.5 mg·kg^−1^ caffeine chewing gum, mouth strips, and a capsule ingestion on 5-km running performance in trained athletes, with a non-significant trend for a greater response following capsule ingestion. Similarly, both coffee mouth rinsing and caffeinated chewing gum over a similar range of doses improved aerobic treadmill running performance of table-tennis players [32]. While both studies offer important insight, the findings are subject to important limitations. Firstly, in the work by Whalley, Dearing and Paton [31], the ingestion period for each mode was matched at 15 minutes prior to exercise which may fail to account for the mode-specific timeframe where peak plasma concentration occurs. More significantly, in both previous studies, performance in caffeine trials were compared to a single capsule placebo trial and therefore participants were not blinded to all the caffeine treatments. Therefore, the demonstrated effects may be influenced by caffeine expectancy, where the belief that consuming caffeine alone is effective in inducing a performance-enhancing effect [33]. Work is needed to directly compare different modes of caffeine supplementation to matched placebos and considering effects on skeletal muscle function where caffeine effects have been suggested to be more ambiguous [9].

To address these knowledge gaps and provide further understanding regarding the effectiveness of alternative modes of caffeine administration, the present work uniquely compared the effect of 3 mg·kg^−1^ caffeine delivered in anhydrous form via capsule ingestion, chewing gum or mouth rinsing on measures of muscular strength, power, and strength endurance in male Rugby Union players. Given that acute effects of caffeine are muscle and contractile mode specific [34], the present study examined effects of force-time characteristics of Countermovement Jump (CMJ), Drop Jump (DJ), Isometric Mid-Thigh Pull (IMTP) performance, and Repetitions until Failure (RTF) of both upper and lower body resistance exercises. It was hypothesized: i) Irrespective of the mode of administration, caffeine would promote enhanced performance, ii) Caffeine anhydrous in capsule form and caffeinated chewing gum would elicit greater effects than caffeine mouth rinsing.

## 2. Materials and methods

Following ethics approval from the host institute [P113453] and written informed consent, 30 participants from the Coventry University Men’s Rugby Union agreed to take part in the study. Sample size estimation was determined using an a priori power calculation (G*power V3.1.9.7; (power: 0.80, alpha: 0.05, effect size: 0.21) for a two factor (Treatment & Mode) repeated measures ANOVA. Previous studies demonstrating performance enhancing effects of 3 mg.kg^−1^ caffeine (administered in capsule form) on measures of muscular strength and power report effect sizes that commonly range between 0.21 and 0.50 [35–37]. Analysis revealed that *n* = 26 participants would be sufficient, with *n* = 30 recruited to account for attrition.

Participants completed a health screen questionnaire and were excluded if they were consuming psychoactive medication, were recovering from or had sustained a musculoskeletal injury in the last six months that was not fully rehabilitated or had underlying contradictions to exercise. Three participants dropped out due to illness (*n* = 1) or inability to attend all scheduled experimental visits (*n* = 2) leaving a final sample of 27 (Mean ± SD Age (yrs) 20 ± 2; Height (cm) 182.0 ± 8.2; Body Mass (kg) 96.6 ± 18.2). Typical average daily caffeine consumption was 188 ± 88 mg as determined using the survey developed by Shohet and Landrum [38]. Eight participants reported no caffeine use.

**Figure 1.**
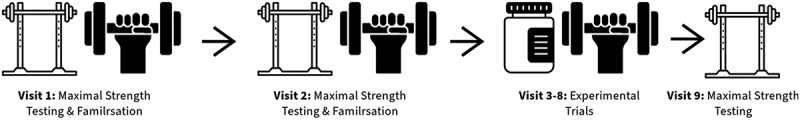
Schematic of experimental approach.

Participants visited the Human Performance Laboratory at the host institute on nine occasions (Figure 1) with each visit separated by a minimum of three but a maximum of five days. Participants were asked to abstain from caffeine and intense physical activity 12hrs and 24hrs prior respectively. All assessments were conducted at the same time of day and participants asked to maintain the same diet and sleep pattern prior to each visit. During the first visit, one repetition maximum (1RM) assessments were completed, and participants were then familiarized to the procedures to be used in the experimental trials. This was repeated in the second visit. In the subsequent six experimental visits, the acute effects of three modes of caffeine were assessed using a double-blind, randomized, and counterbalanced within-subject design. In the final visit, 1RM was reevaluated to determine potential training effects from completion of multiple trials. All sessions took place within the regular season and replaced strength and conditioning sessions. As such, participants had prior experience with several assessments used in the study.

During experimental trials, participants received a 3 mg.kg^−1^ dose of either caffeine anhydrous in capsule form (Caff_CAP_), caffeinated chewing gum (Caff_GUM_), or a caffeine mouth rinse (Caff_RINSE_). Each mode was matched with an identical mode placebo (Plac). Caff_CAP_ was prepared in a single vegetarian capsule (Bulk^TM^, UK) filled with caffeine anhydrous (Bulk^TM^, UK). Plac_CAP_ were prepared in the same way using 3 mg.kg^−1^ maltodextrin (Bulk^TM^, UK). Caff_RINSE_ contained caffeine anhydrous and 3 mg.kg^−1^ sucralose (Bulk^TM^, UK) diluted in 20 ml of water and 30 ml of double concentrated sugar-free orange cordial (Sainsbury’s, UK). Plac_RINSE_ was prepared in the same way without the inclusion of caffeine. Caff_GUM_ treatments were prepared using Healthspan Elite Kick−Start Caffeine Gum (Healthspan Ltd, UK). Each piece of gum has a mass of 2.013 g and contains 100 mg of caffeine. Given an assumed mass:dose ratio of .0497 mg caffeine per 1 mg of gum, treatments were prepared so that each participant received a mass of gum equating to 3 mg.kg^−1^. Plac_GUM_ contained a similar mass of non-caffeinated chewing gum (Mentos, UK). A pestle and mortar were used to grind both Caff_GUM_ and Plac_GUM_ into a single bolus and each treatment was placed in an opaque container where visual inspection of treatments was prohibited.

### 2.1. Maximal strength testing

Participants completed chest press (CP), shoulder press (SP), Deadlift (DL), and Squat (SQ) 1RM as per procedures outlined in our previous work [39]. Participants completed a warm-up consisting of static and dynamic stretching followed by 8–10 repetitions using a 20 kg Eleiko bar (Pullum Power Sports, Luton, UK). Exercises were completed in the following order CP, DL, SP, then SQ. 1RM was determined by progressively increasing mass lifted until the participant failed to complete the lift through a full range of motion and/or technique did not correspond to guidelines outlined by Baechle and Earle [40]. Exercises alternated between upper and lower body to reduce fatigue with maximum weight lifted (kg) recorded once 1RM was achieved. Participants rested for one minute between attempts and five minutes between lifts.

### 2.2. Maximal strength reassessment & familiarization

Participants removed shoes and heavy clothing and measures of height (cm) and body mass (kg) were taken using a portable stadiometer (SECA 213, Hamburg, Germany) and electronic weighing scales (SECA 803, Hamburg, Germany). 1RM re-test was then completed following the protocol previously outlined. 1RM for each exercise was used to determine 70% of 1RM for use in experimental trials. Participants were then familiarized to the assessments used in the experimental trials. Given acute effects of caffeine on muscle function and been suggested to elicit contractile mode and muscle specific [34], a range of assessments were used which have been shown to be highly reliable between sessions [41,42]. Furthermore, many of the assessments are regularly employed monitoring and screening tools [43].

### 2.3. Countermovement jump

Bilateral CMJ performance was quantified using two Hawkin Dynamics force platforms (Hawkin, Maine, USA) sampling at 1000 hz. With arms akimbo and following a period of quiet standing, participants were instructed to “*jump as high and as fast as possible*.” All participants completed three successful jumps with 60s rest between attempts. Using the attempt that elicited the greatest jump height (cm), contact time (ms), RSI (Reactive Strength Index; jump height (m)/contact time (ms)) and phase specific force-time metrics were determined to provide insight into both vertical jump performance and strategy.

### 2.4. Drop jumps

Bilateral DJ performance was also quantified using performed two Hawkin Dynamics force platforms (Hawkin, Maine, USA) sampling at 1000 hz. Participants stood upright on box positioned 40 cm above the force platforms. With arms akimbo and following a period of quiet standing, participants were asked to step off the box with their dominant leg, land bilaterally and immediately “*jump as high and as fast as possible*.” Participants completed three successful attempts separated by 60s rest. Jumps were discounted if participants stepped down or jumped upwards of the box, if feet did not land simultaneously, or if foot position crossed force platforms on the second landing. Using the jump that elicited the greatest RSI, jump height (cm), contact time (ms), and phase specific force-time metrics were determined.

### 2.5. Isometric mid-thigh pull

IMTPs were performed on two floor mounted triaxle force platforms (AMTI, ACP, Waterton, MA) sampling at 1000 hz and using a custom-built steel rack fixed to the ground. In accordance with procedures outlined by Comfort, Dos’Santos [44], participants were asked to stand above the bar with a knee angle of 135–145° and a hip angle of 140–150°. Participants used lifting straps to reduce the loss of grip. The bar height used by each participant was retained for subsequent assessments. Participants completed three 3s warm-up trials at 50% and 75% of their perceived maximal effort prior to measured attempts. Using minimal pretension and following a minimum one second period where the force data trace was stable, participants were instructed to “*push your feet into the ground as hard and as fast as possible*,” for a duration of ~ 5s. Participants completed three attempts separated by 2-minute rest. Raw unfiltered F_z_ data were extracted for analysis and PF (N.kg), PF (N.kg) at 100 ms (F100) and 300 ms (F300) were determined in accordance with recommend procedures [44].

### 2.6. Resistance exercise repetitions until failure

Participants completed RTF assessments of CP, SP, SQ, and DL. Participants completed two sets at 70% of 1RM as per previous work [36]. A trained spotter was present during all resistance exercises ensuring proper range of motion and any lift that deviated from guideline outlined by [40] was not counted toward total repetitions completed. Exercises were completed in the following order: CP, DL, SP, then SQ altering from upper to lower body. A minimum of 2-minute rest was permitted between exercises and a 10-minute rest between the first and second sets. Total repetitions completed were recorded post completion of each exercise and Rating of Perceived Exertion (RPE) assessed using the 20-point Borg scale [45].

### 2.7. Experimental trials

**Figure 2.**
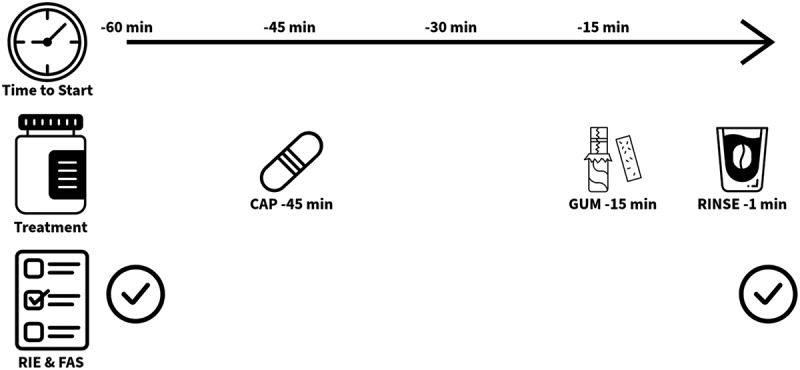
Schematic of treatment ingestion period for all caffeine modes of administration [RIE = readiness to invest effort, FAS = felt arousal scale].

Treatment periods were standardized to 60 minutes prior to the initiation of exercise were administered in a mode specific timeframe (Figure 2). Caff_CAP_ was ingested with 150 ml of water 45 minutes prior to exercise given that peak plasma concentration typically occurs between 30 and 60 minutes post consumption [46]. Caff_GUM_ was administered 10 minutes prior to exercise, chewed for 5 minutes allowing the commencement of exercise to occur within the timeframe of the first peak in caffeine plasma concentration [14]. Caff_RINSE_ was undertaken 1 minute prior to exercise, where participants rinsed the solution around the mouth for 30 seconds and then expectorated the solution into a waste bucket. Plac treatments were administered in the same way. Between arrival and the onset of exercise participants were asked to sit and rest. At arrival and prior to exercise, participants motivation for exercise was measured using the Felt Arousal Scale (FAS) [47] and by completion of the Readiness to Invest Physical (RIPE) and Mental Effort (RIME) scale [48]. Experimental trials followed the procedures outlined above following completion the warm-up previously explained.

### 2.8. Statistical analysis

Statistical analysis was performed using Statistical Package for the Social Sciences (IBM SPSS Statistics Version 28). To detect any potential training effect from continuous bouts of resistance exercise pre- and post-experimental 1RM performance was evaluated using a paired t-tests. To determine the effects of caffeine, RIPE, RIME and FAS data were assessed using a 3-factor repeated-measures ANOVA with Treatment (Caff or Plac), Mode (capsule, gum, or rinse) and Time (pre-and post-ingestion) as factors. Acute effects of caffeine on CMJ, DJ, and IMTP were analyzed using a 2-factor repeated measure ANOVA with Treatment (Caff or Plac) and Mode (capsule, gum, or rinse) as factors. RTF and RPE were analyzed using a 3-factor repeated-measures ANOVA with Treatment (Caff or Plac), Mode (capsule, gum, or rinse), and Set (set 1 or set 2) as factors. For ANOVA, Greenhouse-Geisser adjustment was interpreted on occasions where sphericity was violated, and relevant significant main effects and interactions were explored via Bonferroni adjusted pairwise comparisons. For ANOVA, Partial eta squared (ηp^2^) as a measure of effect size and categorized as small (0.01), medium (0.06), and large (0.14) [49]. For pairwise comparison and t-tests, Cohen’s d corrected for bias using Hedge’s g was determined and interpreted as trivial <0.20, small 0.20–0.49, medium 0.50–0.79, and large > 0.80 [50]. Data are presented as Mean ± SD with statistical significance set at *p* < 0.05.

## 3. Results

Table 1 summarizes the effect of caffeine on pre-exercise perceived motivation. For RIPE there was a Treatment*Mode*Time interaction (*p* = .038; ηp^2^=.118). Pairwise comparisons indicated that RIPE was higher Pre exercise in Caff_GUM_ compared to Plac_GUM_ (*p* = .039; d = 418), that RIPE was higher in the Caff_RINSE_ trial both pre and post ingestion compared to Plac_RINSE_ (*p* < 0.022; d > .470). Irrespective of treatment and mode, RIPE was increased from pre ingestion to pre-exercise (*p* < .001; d > .826).

**Table 1.** Acute effect of 3 mg.Kg-1 Caff_CAP_, Caff_GUM_ and Caff_RINSE_ on RIPE, RIME and FAS.

		Plac_CAP_	Caff_CAP_	d=	Plac_GUM_	Caff_GUM_	d=	Plac_RINSE_	Caff_RINSE_	d=
RIPE	Pre-Ing	4.2 ± 2.0	4.7 ± 2.4	0.24	4.4 ± 2.1	4.4 ± 2.1	0.02	4.1 ± 2.0	5.3 ± 2.1	0.56
	Pre-Ex	5.9 ± 2.1	6.1 ± 2.1	0.09	5.4 ± 2.0	6.1 ± 1.7	0.42	5.4 ± 1.8	6.4 ± 1.9	0.47
RIME	Pre-Ing	4.4 ± 2.1	5.0 ± 2.3	0.29	4.9 ± 2.1	4.8 ± 2.2	0.04	4.5 ± 1.9	5.4 ± 2.1	0.34
	Pre-Ex	6.3 ± 2.0	6.8 ± 1.6	0.23	6.2 ± 2.0	6.7 ± 1.7	0.34	6.5 ± 1.7	6.8 ± 1.7	0.15
FAS	Pre-Ing	3 ± 1	3 ± 1	0.11	3 ± 1	3 ± 1	0.07	3 ± 1	3 ± 1	0.38
	Pre-Ex	4 ± 1	3 ± 1	0.35	3 ± 1	3 ± 1	0.03	4 ± 1	3 ± 1	0.11

Values are represented as means ± SD, Plac = Placebo, Caff = Caffeine, CAP = Capsule, RIPE = Readiness to Invest Effort Physical, RIME = Readiness to Invest Effort Mental, FAS = Felt Arousal Scale, Pre-Ing = Pre-ingestion, Pre-ex = Post-Exercise d = Effect Size.

For RIME there were no significant interactions (*p* > .050: ηp^2^<.110) and no main effect of mode (*p* = .802: ηp^2^=.008). There were however significant main effects of both treatment (*p* = .038: ηp^2^=.156) and time (*p* = .001: ηp^2^=.765) where caffeine treatment increased RIME and RIME was increased from pre ingestion to pre-exercise.

For FAS there were significant interactions (*p* > .128: ηp^2^<.077) and no main effects of Treatment (*p* = .154: ηp^2^=.077) or Mode (*p* = .450: ηp^2^=.077). There was however a main effect of Time (*p* < .001: ηp^2^=.834), indicating that FAS was increased from pre-ingestion to pre-exercise.

Table 2 summarizes the performance data for the CMJ. For jump height there was a main effect of treatment (*p* = .035; ηp^2^=.160), indicating that performance in the caffeine trials was higher than in the placebo trials. There was no main effect of mode (*p* = .688; ηp^2^=.014) or treatment*mode interaction (*p* = .582; ηp^2^=.021). Similarly, RSI was higher following caffeine treatment at a level that was approaching critical alpha and with a large effect size (*p* = .0.053; ηp^2^=.137). However, there was no main effect of mode (*p* = .351; ηp^2^=.004) or treatment*mode interaction (*p* = .659; ηp^2^=.016). There was no main effect of treatment (*p* > .087; ηp^2^<.108), mode (*p* > .444; ηp^2^<.032) or a treatment*mode interaction (*p* > .057; ηp^2^<.105) for any of the remaining CMJ force-time variables.

**Table 2.** Acute effect of 3 mg.Kg-1 Caff_CAP_, Caff_GUM_ and Caff_RINSE_ on CMJ performance.

	Plac_CAP_	Caff_CAP_	d=	Plac_GUM_	Caff_GUM_	d=	Plac_RINSE_	Caff_RINSE_	d=
Jump Height (cm)*	31.9 ± 5.8	32.6 ± 6.3	0.15	31.5 ± 6.7	33.3 ± 6.7	0.39	31.3 ± 5.6	32.3 ± 6.9	0.24
RSI	0.36 ± 0.08	0.38 ± 0.1	0.24	0.38 ± 0.11	0.39 ± 0.1	0.09	0.36 ± 0.07	0.38 ± 0.1	0.33
Contact Time (ms)	892.1 ± 122.8	874 ± 153.3	−0.12	848.3 ± 125.3	887.7 ± 159.0	0.4	886.7 ± 107.0	871.6 ± 117.2	−0.11
Unweighting Time (ms)	427.3 ± 80.7	410.1 ± 112.8	−0.15	377.1 ± 82	426.7 ± 106.6	0.57	418.7 ± 93.3	401.9 ± 69.4	−0.14
Breaking Time (ms)	196 ± 46.8	192.2 ± 41.2	−0.11	200.9 ± 41.8	189.7 ± 39.6	−0.38	197 ± 46.2	198.2 ± 38.8	0.04
Depth (cm)	−29.9 ± 7.3	−30 ± 7.4	−0.02	−29.7 ± 6.4	−30.8 ± 7.2	−0.28	−29.6 ± 6.7	−30 ± 7.1	−0.10
Force @ 0 Velocity (N)	1929.4 ± 332.3	1980.5 ± 398.7	0.25	1941.8 ± 413.0	2020.1 ± 365.9	0.25	1960.2 ± 373.7	1932.2 ± 342.5	−0.16
Breaking RFD (N/s)	5612.7 ± 2077	5971.9 ± 2477.4	0.19	5444.4 ± 2312.6	6120.5 ± 2194.7	0.41	5604.9 ± 2102.8	5472.4 ± 2097.8	−0.10
Breaking Net Impulse (N.s)	97 ± 23.9	101.4 ± 29.2	0.32	97.1 ± 24.9	101.8 ± 29.7	0.21	100.8 ± 26.9	97.5 ± 24.8	−0.25
Mean Breaking Power (Watts)	−929.4 ± 253.5	−993.6 ± 336.1	−0.37	−930.1 ± 271.9	−1003.6 ± 328.0	−0.29	−966.7 ± 294.4	−932.4 ± 262.1	0.20
Propulsive Time (ms)	268.8 ± 40.2	271.7 ± 43.7	0.10	270.3 ± 38.4	271.3 ± 40.7	0.04	271.1 ± 43.7	271.6 ± 40.1	0.02
Propulsive Net Impulse (N.s)	231.3 ± 38.4	233.3 ± 39.8	0.15	230.5 ± 41.4	238.9 ± 41.1	0.32	232.2 ± 37.6	232.1 ± 37.3	0.00
Mean Propulsive Power (Watts)	2425.7 ± 428.2	2476.8 ± 512.7	0.19	2419.2 ± 506.9	2534.3 ± 474.5	0.37	2432 ± 449.9	2432.8 ± 429.4	0.01

Values are represented as Mean± SD, Plac= Placebo, Caff= Caffeine, CAP=Capsule, d = Effect Size. * demonstrates main effect of treatment.

Table 3 summarizes the data for the DJ. For jump height and each of the force-time variables measured there was no main effect of treatment (*p* > .332; ηp^2^<.0.04), mode (*p* > .177; ηp^2^<.065) or a treatment*mode interaction (*p* > .350; ηp^2^<.040).

**Table 3.** Acute effect of 3 mg.Kg-1 Caff_CAP_, Caff_GUM_ and Caff_RINSE_ on DJ performance.

	Plac_CAP_	Caff_CAP_	d=	Plac_GUM_	Caff_GUM_	d=	Plac_RINSE_	Caff_RINSE_	d=
Jump Height (cm)	20.8 ± 10	19.8 ± 9	−0.11	20.1 ± 7.8	20.2 ± 8.1	0.01	17.8 ± 7.3	19.6 ± 6.7	0.29
RSI	0.42 ± 0.23	0.39 ± 0.19	−0.19	0.39 ± 0.17	0.39 ± 0.16	−0.02	0.35 ± 0.17	0.38 ± 0.14	0.22
Contact Time (ms)	532.3 ± 120	532.0 ± 91.9	0.00	535.9 ± 91.2	542.2 ± 108.7	0.09	525.7 ± 77.9	532.4 ± 96.9	0.09
Breaking Time (ms)	296.8 ± 87.6	297.0 ± 68.8	0.01	305.2 ± 68.9	307.8 ± 78.3	0.04	305.0 ± 69.0	304.5 ± 88.3	−0.01
Breaking Net Impulse (N.s)	263.1 ± 48.2	264.5 ± 48.9	0.12	263.6 ± 50	263.9 ± 48.6	0.02	268.9 ± 49.2	264.6 ± 50.9	−0.38
Mean Breaking Power (Watts)	−2773.6 ± 516.1	−2789 ± 556.5	−0.05	−2717 ± 493	−2720.3 ± 505.8	−0.01	−2806.3 ± 591.0	−2740.4 ± 538.7	0.21
Propulsive Time (ms)	235.5 ± 53.1	235.0 ± 45.6	−0.01	230.7 ± 45.2	234.4 ± 54.8	0.11	220.7 ± 40.8	227.9 ± 50.8	0.18
Propulsive Net Impulse (N.s)	399.9 ± 100.8	396.3 ± 92.1	−0.06	394.7 ± 93.6	397.3 ± 91.3	0.05	382.3 ± 93.3	388.6 ± 99.4	0.09
Mean Propulsive Power (Watts)	1925 ± 600	1880.5 ± 549.5	−0.11	1925.8 ± 545.6	1913.1 ± 485.7	−0.03	1846.1 ± 556.2	1876.9 ± 483.4	0.07

Values are represented as Mean± SD, Plac= Placebo, Caff= Caffeine, CAP=Capsule, RSI = Reactive Strength Index, d = Effect Size.

Table 4 summarizes the data for the IMTP. For peak force and force measured at 100 ms and 300 ms following the initiation of the pull there was no main effect of treatment (*p* > .586; ηp^2^<.013) or a treatment*mode interaction (*p* > .317; ηp^2^<.066). There was no main effect of mode (*p* > .061; ηp^2^<.103), other than for force measured at 300 ms (*p* = .039; ηp^2^<.117). However, pairwise comparison demonstrated no difference between modes (*p* > .08; *d* < .35).

**Table 4.** Acute effect of 3 mg.Kg-1 Caff_CAP_, Caff_GUM_ and Caff_RINSE_ on IMTP performance.

	Plac_CAP_	Caff_CAP_	d=	Plac_GUM_	Caff_GUM_	d=	Plac_RINSE_	Caff_RINSE_	d=
Peak Force (N/kg)	28.9 ± 5.7	29.7 ± 4.5	−0.19	28.6 ± 5.4	28.9 ± 4.9	0.08	27.8 ± 4.8	29.1 ± 4.8	0.39
Force (N/kg) @ 100 ms	14.8 ± 2.6	15.2 ± 2.9	−0.10	13.4 ± 2.4	14.6 ± 2.8	−0.46	14.3 ± 2.3	14.4 ± 2.5	−0.06
Force (N/kg) @ 300 ms	22.2 ± 3.3	22.4 ± 3.2	−0.06	21.8 ± 4.1	22.4 ± 3.1	−0.16	21.0 ± 3.0	21.2 ± 4.5	0.04

Values are represented as Mean± SD, Plac= Placebo, Caff= Caffeine, CAP=Capsule, RSI = Reactive Strength Index, d = Effect Size.

Table 5 summarizes RTF performance and post set RPE data. For RTF for the DL there were no significant interactions (*p* > .501; ηp^2^<.027), and no main effect of treatment (*p* = .373; ηp^2^=.03) or mode (*p* = .248; ηp^2^=.052). The number of DL completed was reduced in the second set (*p* < .001; ηp^2^=.799). Similarly for CP there were no significant interactions (*p* > .566; ηp^2^<.023), and no main effect of treatment (*p* = .832; ηp^2^=.002). There was, however, a main effect of both mode (*p* = .012; ηp^2^=.155) and set (*p* < .001; ηp^2^=.899), indicating the number of CPs was reduced in the second set and that the number of repetitions was greater in the rinse trials compared to the capsule trials (*p* = .014; d = .439).

**Table 5.** Acute effect of 3 mg.Kg-1 Caff_CAP_, Caff_GUM_ and Caff_RINSE_ on resistance exercise repetitions until failure and post set RPE.

		Plac_CAP_	Caff_CAP_	d=	Plac_GUM_	Caff_GUM_	d=	Plac_RINSE_	Caff_RINSE_	d=
*Repetitions Until Failure*										
CP	Set1	13 ± 3	14 ± 2	0.1	14 ± 2	14 ± 3	0.04	15 ± 3	15 ± 3	0.06
	Set2	11 ± 3	11 ± 2	0.08	12 ± 3	12 ± 3	0.03	12 ± 2	12 ± 2	0.05
SP	Set1	11 ± 3	13 ± 3	0.39	11 ± 3	11 ± 3	0.04	11 ± 3	12 ± 3	0.44
	Set2	9 ± 2	10 ± 3	0.3	9 ± 3	10 ± 2	0.02	9 ± 3	10 ± 3	0.27
SQ	Set1	13 ± 5	14 ± 5	0.41	13 ± 3	14 ± 4	0.45	13 ± 3	14 ± 3	0.66
	Set2	11 ± 3	12 ± 4	0.37	11 ± 4	11 ± 3	0.1	11 ± 3	11 ± 4	0.06
DL	Set1	12 ± 4	12 ± 3	0.16	12 ± 4	12 ± 3	0.11	12 ± 4	12 ± 4	0.04
	Set2	9 ± 3	10 ± 3	0.27	10 ± 3	10 ± 3	0.01	10 ± 4	10 ± 3	0.06
*Post Set RPE*										
CP	Set1	18 ± 1	18 ± 1	0.19	18 ± 1	18 ± 1	0.09	18 ± 1	18 ± 1	0.06
	Set2	19 ± 1	19 ± 1	0.05	19 ± 1	19 ± 1	0.16	18 ± 2	18 ± 1	0.05
SP	Set1	17 ± 1	18 ± 1	0.13	17 ± 1	17 ± 1	0.03	17 ± 1	17 ± 1	<.001
	Set2	19 ± 1	19 ± 1	0.03	19 ± 1	19 ± 1	0.14	19 ± 1	19 ± 1	0.11
SQ	Set1	17 ± 1	18 ± 1	0.66	17 ± 1	17 ± 1	0.12	18 ± 1	18 ± 1	0.36
	Set2	19 ± 1	19 ± 1	0.08	19 ± 1	19 ± 1	0.06	19 ± 1	19 ± 1	0.35
DL	Set1	18 ± 1	18 ± 1	0.23	18 ± 1	18 ± 1	0.05	18 ± 1	18 ± 1	0.28
	Set2	19 ± 1	19 ± 1	0.13	19 ± 1	19 ± 1	0.12	19 ± 1	19 ± 1	0.28

Values are represented as means ± SD, Plac= Placebo, Caff= Caffeine, CAP=Capsule, CP= Chest Press, SP= Shoulder Press, SQ= Squats, DL= Deadlift, d = Effect Size.

For SP, there were no interactions (*p* > .280; ηp^2^<.047) other than for Mode*Set (*p* < .020; ηp^2^=.140). Pairwise comparisons indicated that when each set was compared, there was no effect of mode (*p* > .226; d < .263). For each mode, the number of reps completed was reduced in the second set (*p* < .001; d > 1.069). There was also no main effect of treatment (*p* = .104; ηp^2^=.002).

For SQ, there were no interactions (*p* > .333; ηp^2^<.042) other than for Treatment*Set (*p* < .001; ηp^2^<.360). Pairwise comparisons indicated that caffeine treatment increased the number of reps completed in the first set (*p* < .001; d = .481) but not the second set (*p* = .133; d = .136). There was no main effect of mode (*p* = .902; ηp^2^<.004).

CP, SP and DL there were no significant interactions (*p* > .496; ηp^2^<.028), and no main effects of treatment (*p* > .280; ηp^2^<.047) or mode (*p* > .185; ηp^2^<.064) for measures of RPE. There was a main effect of set (*p* > .280; ηp^2^<.047), demonstrating that RPE was higher following completion of the second set.

For SQ there was a Treatment*Mode*Set interaction (*p* = .009; ηp^2^=.165). Pairwise comparisons indicated that upon completion of the first set, RPE was higher in Caff_CAP_ compared to Plac_CAP_ (*p* = .002; d = .663), there were no other treatment effects (*p* > .069; d < 365). RPE following completion of set one was higher in Plac_RINSE_ compared to Plac_CAP_ and Plac_GUM_ (*p* < .044; d > .503). Irrespective of mode or treatment, RPE was higher following the second set compared to the first set (*p* < .001; d > 1.05).

## 4. Discussion

The present study uniquely compared the effectiveness of 3 mg.kg^−1^ caffeine administered via a capsule, gum or mouth rinse on the muscular strength and power of university standard Rugby Union players. Irrespective of mode of administration, there were limited effects of caffeine compared to a mode matched placebo across the breadth of strength and power outcomes measured. Caffeine did however elicit a small, but significant, increase in CMJ height, SQ RFT and pre-exercise RIME, with no interaction between treatment and mode demonstrating equivalent effects across modes of administration. Results of the present study therefore demonstrate that novel modes of caffeine administration proposed to evoke benefits via distinct mechanisms do not offer unique effects and given the small number of performances enhancing benefits, athletes participating in multimodal sports should carefully consider the strength and limitations of acute caffeine consumption for the purpose of improving sports performance.

Several meta-analyses have demonstrated small but significant benefits of caffeine anhydrous ingestion on measures of maximal strength, power and rate of force development [1,2,51,52], and while benefits for CMJ height and SQ performance demonstrated in the present study would appear in keeping with this, the present results fail to demonstrate caffeine-induced effects on several other strength and power outcomes. This is not unusual, with a number of studies, inclusive of those incorporated in meta-analyses, failing to demonstrate effects of acute caffeine ingestion [53–55]. Several factors, such as the potential of muscle and contractile mode specific effects, dose administered, habituation, training status and difference in gene polymorphisms responsible for caffeine metabolism and sensitivity, have been suggested moderators to caffeine effects and used to explain equivocal findings [56]. Moreover, in a number of cases the general acceptance that acute caffeine consumption may be beneficial for strength and power performance is based on previous work, inclusive of our own, that draws conclusions from effects specific to only some of the included measures [57–59], or from studies that fail to comprehensively consider mechanically distinct strength and power assessments. A particular strength of the present study is the broad range of measures utilized that incorporate a range of contractile mechanics. However, considering only a small number of positive effects, it would be an overstatement to summaries that caffeine is beneficial for muscular strength and power performance.

A caffeine induced increase in CMJ height is in keeping with findings summarized in a recent meta-analysis [60] and given a strong association with lower body power skills [61], may be important to sports performance. However, the translation of a relatively small performance benefit to biomechanically more complex movements utilized in team sports is not clear. Despite several studies quantifying the effects of acute caffeine consumption of CMJ performance, with only few exceptions [62], there is a distinct lack of studies that have attempted to characteristics the force-time characteristics, and the small number of studies that have considered this do not provide a comprehensive approach [37,63]. Whist jump height allows us to determine if the performance outcome is improved, force-time characteristics provide important insight into understanding how the performance outcome was achieved. The lack of effect on the force-time characteristics measured in the present study would appear to undermine the caffeine induced performance benefit, however, these data more likely indicate challenges with associating small increases in jump performance with specific force-time metrics and that enhanced performance may be explained by a series of small non-significant increases in a number of the measured force-time outcomes (i.e. braking phase metrics in the case of Caff_GUM_).

In comparison to the CMJ, there was no effect of caffeine on DJ performance in the present study. Studies examining the effects of caffeine on DJ performance are sparse and these data indicate that parity of caffeine effects across measures of vertical jump performance should not be assumed. Biomechanically DJ performance may have greater relevance to sports specific tasks. The ability to produce power rapidly following a period of deceleration may be more representative of the mechanical constraints placed on athletes. However, it should be considered that the lack of effect demonstrated in the present study may be specific to the drop height used and the capability of participants to be able to complete the task. While a 40 cm drop height is not uncommon in drop landing tasks, this height appears to exceed the height for optimal deceleration and stretch shortening cycle mechanics, as evidenced by the low RSI and long contact time of the participants. This indicates that the drop height provided a substantial eccentric challenge for center of mass declaration and results may not translate to DJ completed at a lower height that allows optimal stretch shortening cycle function.

A lack of caffeine induced effects on IMTP performance is in keeping with previous work and the effect specific to SQ in the RTF protocol underpin the complexity in understanding caffeine’s effect on muscular strength and power. The lack of consistent effects may be explained by the muscle group and contractile mode specific nature of the caffeine effect [34] and that small effects from supplementation are difficult to detect [36].

Given the limited caffeine induced effects, the present work fails to offer further support to the effectiveness of alternative modes of caffeine administration for eliciting improved muscular strength and power performance. Mouth rinsing and maceration of caffeine gum in the mouth does not appear to elicit either unique or superior effects. While the results for Caff_RINSE_ are somewhat in keeping with results from studies that have examined the effects of a single Caff_RINSE_ on physical performance [30], the findings are at odds with the growing evidence supporting the ergogenic potential of Caff_GUM_ [22]. However, the lack of research specific to strength and power assessment should be acknowledged. Given differences in exercise modalities, and the use of mode matched placebos, it is difficult to make direct comparisons between the results of the present study and that of previous work that has compared the effects of different caffeine modes [31,32]. Even on the small number of occasions when a caffeine effect was demonstrated (CMJ, height, SQ RTF and RPE, pre-exercise RIME), there were no other outcomes determined by the results of the statistical test that indicated effects favored any particular mode. While this might be interpreted as equivalent effects of the different caffeine modes, which may indeed be the case for SQ RTF given the moderate effect size measured when each caffeine mode was compared to a mode matched placebo, equivalent effects appear unlikely in the other measures where a main effect of caffeine was demonstrated given that effect sizes for placebo matched comparisons ranged from trivial to small. Despite mode matched placebos being a particular strength of our study design, the ANOVA conducted is somewhat limited in this sense given that the main effect of caffeine represents an amalgamation of the three caffeine trials, indicating that caffeine may elicit small benefits across the three trails that may not be evidence on a single occasion. This again questions the practical relevance of these small number of effects and supports the basis to conclude that caffeine has limited acute performance enhancing benefit in this context.

### 4.1. Limitations & implications

While this study offers unique insight into the effects of different modes of caffeine administration on muscular strength and power, it is not without limitation. Importantly, gene polymorphisms involved with caffeine metabolism (CYP1A2) and sensitivity (ADORA2A), dose, and training status have been suggested to moderate caffeine effects [64] and were not measured in this study and are also distinct limitations of the majority of prior work. However, there is still difficulty in directly attributing ergogenic effects to specific gene polymorphisms given several studies showing no association [65]. Studies examining the association between the performance enhancing effects of caffeine and ADORA2A genotypes are lacking [56] and data supporting an association with the CYP1A2 genotype are typically specific to endurance exercise [66], and the weight of supporting evidence drawn from studies with a reported conflict of interest [67]. While an optimal genotype profile may exist, the systemic effects of caffeine mean that examining and attributing effects to a single gene polymorphism is currently still somewhat limited. While a need for future work is evident, it would appear valuable to extend this to understanding the association between specific genotypes and the potential for a performance enhancing response elicited by different modes of caffeine administration given the potential for unique mechanistic effects.

Although it is generally accepted that there is no dose-response effect, there is evidence suggesting that the prevalence of caffeine effects on some measures of muscular strength may only be detectable at higher doses [34]. While recent studies have evaluated and demonstrated effects of 3 mg.kg^−1^ caffeine on measures of muscular function, the wealth of supporting evidence is specific to caffeine administered at higher doses (typically 5–6 mg.kg^−1^) [52]. 3 mg.kg^−1^ is more representative of doses consumed by athletes and that achievable from consumption of commercially available products, and therefore, results of the present study offer further important insight into the ergogenic potential of caffeine at this concentration. However, results of the present study may not directly extrapolate to higher caffeine doses, and future work should consider examining mode specific responses at 6 mg.kg^−1^ in light of the results of a recent meta-analysis indicating that such doses may elicit greater effects on measures of muscular strength compared to lower doses (2–5 mg.kg^−1^ considered as the low dose group) [52]. Although at present, providing 6 mg.kg^−1^ in the form of caffeinated gum would present a significant challenge given the large bolus that would be required due to the relatively low dose provided form this mode of administration. For example, over five pieces of the caffeinated gum used in the present study would need to be provided to a participant with a body mass of 85 kg to achieve 6 mg.kg^−1^ dose.

To examine the effect of different caffeine delivery vehicles, a particular strength of the study design was to use a matched caffeine dose. Although batch checked HelathSpan caffeine gum was used, gum was administered to participants by mass and based on an assumed equal caffeine distribution in each piece. While this is unlikely to be the case, the effects on the final delivered does are likely to be minimal given that this was only required for a proportion of the total gum mass administered (i.e. that exceed values divisible by 100 mg (i.e. one full piece of gum)).

With respect to practical implications of our data, it may be conceived that even the potential for small increase in performance that requires minimal effort may position caffeine as a suitable low risk: high reward nutritional strategy for team sports athletes. However, practitioners may need to exercise caution when administering caffeine to team sport athletes given that higher doses typically prescribed to induce improved physical performance may negatively impact cognitive function. Moreover, potential caffeine effects should be balanced with the impact on sleep hygiene, impaired mood and exercise recovery, though such effects are yet to be robustly investigated.

## 5. Conclusion

3 mg.kg^−1^ caffeine administered via capsule, gum or mouth rinse had limited effects on the muscular strength, power, and strength endurance of male university standard Rugby Union players. On the small number of occasions where a caffeine effect was prevalent, there was no interaction between treatment and mode, and when effects were directly compared to a mode matched placebo, effects were typically trivial or small indicating limited translation of a beneficial effect of caffeine to a single performance trial. Interestingly, the small effect of caffeine on CMJ height could not be explained by changes in specific ground reaction force-time characteristics and were not transferable to DJ performance, and effects specific to the SQ RTP exercise underpin the complexity in understanding effects of caffeine on muscular function. Collectively, results of the present study indicate that novel modes of caffeine administration proposed to evoke benefits via distinct mechanisms do not offer unique effects with respect to measures of muscular function. Given the small number of performance enhancing benefits, athletes participating in multimodal sports should carefully consider the strength and limitations of acute caffeine consumption for the purpose of improving sports performance.
